# Individual Differences in the Discrimination of Novel Speech Sounds: Effects of Sex, Temporal Processing, Musical and Cognitive Abilities

**DOI:** 10.1371/journal.pone.0048623

**Published:** 2012-11-05

**Authors:** Vera Kempe, John C. Thoresen, Neil W. Kirk, Felix Schaeffler, Patricia J. Brooks

**Affiliations:** 1 University of Abertay Dundee, Dundee, Scotland, United Kingdom; 2 Brain Mind Institute, Ecole Polytechnique Fédérale de Lausanne, Lausanne, Switzerland; 3 University of Abertay Dundee, Dundee, Scotland, United Kingdom; 4 Queen Margaret University Edinburgh, Edinburgh, Scotland, United Kingdom; 5 College of Staten Island and the Graduate Center of City University of New York, Staten Island, New York, United States of America; Utrecht University, The Netherlands

## Abstract

This study examined whether rapid temporal auditory processing, verbal working memory capacity, non-verbal intelligence, executive functioning, musical ability and prior foreign language experience predicted how well native English speakers (N = 120) discriminated Norwegian tonal and vowel contrasts as well as a non-speech analogue of the tonal contrast and a native vowel contrast presented over noise. Results confirmed a male advantage for temporal and tonal processing, and also revealed that temporal processing was associated with both non-verbal intelligence and speech processing. In contrast, effects of musical ability on non-native speech-sound processing and of inhibitory control on vowel discrimination were not mediated by temporal processing. These results suggest that individual differences in non-native speech-sound processing are to some extent determined by temporal auditory processing ability, in which males perform better, but are also determined by a host of other abilities that are deployed flexibly depending on the characteristics of the target sounds.

## Introduction

The ability to process rapidly changing temporal information is considered to be fundamental to the process of identifying speech sounds. For example, temporal processing of auditory information in the range of tens to hundreds of milliseconds (ms) is crucial for identifying consonants, which differ in features such as formant transitions signalling place of articulation or voice onset time signalling the contrast between voiced and voiceless consonants. Neuroimaging and electrophysiological evidence suggests that the detection of rapid temporal changes in auditory stimuli is predominantly associated with neural activation in the left hemisphere in areas that are involved in speech processing [1], [2], [3], [4], [5]. Impairments of temporal auditory processing have been implicated in speech perception disabilities such as dyslexia [6], [7], [8], [9], [10].

**Figure 1:**
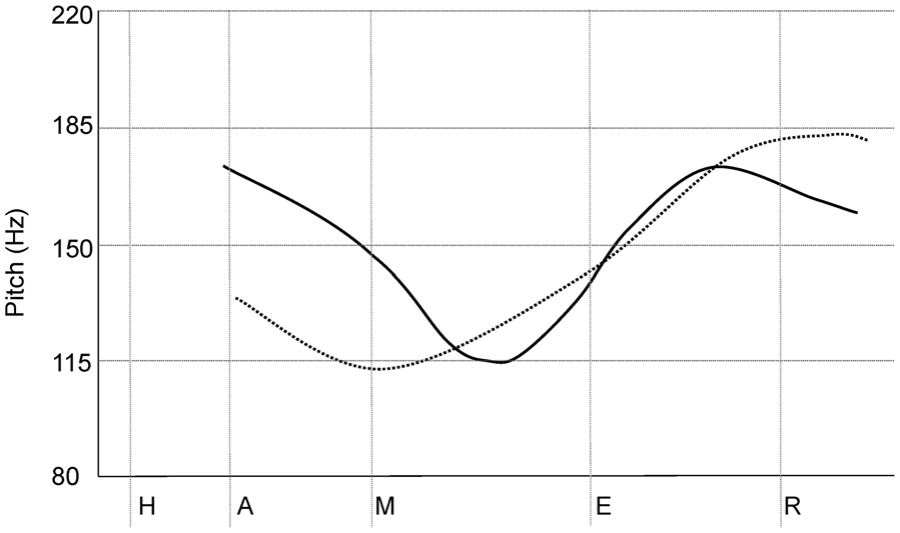
Illustration of the different pitch contours of minimal pairs of Norwegian tones for the homophone/hamer/. The dotted line shows the rising tone for the proper noun ‘Hammer’. The solid line shows the falling-rising tone for the noun ‘hammer’ denoting the tool.

The present study examined the extent to which the ability to process rapidly changing temporal information contributes to individual differences in the processing of non-native speech sounds by healthy adults. We were specifically interested in exploring whether temporal auditory processing might explain a male advantage in non-native speech-sound processing observed in previous studies. Previous research had found a very small but statistically significant male advantage in the processing of a tonal contrast – the contrast between rising and falling-rising Norwegian tones in a sample of 414 adult native English speakers [11]. In many dialects of Norwegian, lexical tone encompasses pitch accents which distinguish minimal pairs of segmentally identical bi-syllabic words, and involves temporal changes in fundamental frequency in the range of several hundreds of milliseconds (see Figure 1). A male advantage was also observed for two Hindi consonant contrasts involving differences in voice onset times in a sample of 1,580 adult native English speakers [12]. In a much smaller sample of only 48 adults, a male advantage was reported for the discrimination of binaurally presented pitch contours of computer-generated waveforms comprising a fundamental frequency and two formants [13]. Thus, sex differences have been observed for the perception of a variety of speech sounds that require temporal processing of auditory stimuli with a time course of change in acoustic parameters ranging from under 100 ms for consonantal contrasts to up to about 300 ms for pitch contours and lexical tones. These sex differences are consistent with studies of non-linguistic temporal processing, which have also shown that men tend to outperform women in temporal order judgments [14], [15] and temporal discrimination tasks involving the detection of changes in the acoustic properties of stimuli in the range of 200 ms [16]. A recent study has also found sex differences in the ability to imitate non-native speech sounds: Reiterer and colleagues [17] showed that men were overrepresented amongst the successful imitators, and under-represented amongst the poor imitators. While a variety of factors have been invoked to explain this finding, the ability to discriminate rapidly changing features of sound may be one of the mechanisms that puts men at an advantage for imitation, given that peception and production abilities are closely linked [18].

Rapid temporal processing is associated with measures of higher-order cognitive functioning such as psychometric intelligence [19] and working memory capacity [20]. However, some studies concerned with the role of perceptual and cognitive factors in native speech processing under adverse conditions, such as noise and hearing loss, have failed to establish a link between rapid temporal processing and measures of general cognitive ability [21], [22], [23]. Instead, it has been suggested that auditory processing abilities and cognitive abilities make independent contributions to native speech processing under adverse conditions [22]. Here, we examine whether higher-order cognitive abilities can explain individual differences, including sex differences, in non-native speech sound processing in healthy adults, and whether their role is mediated by, or independent from, rapid temporal auditory processing.

To address this question, we presented native speakers of English with the Norwegian tonal contrast between rising and falling-rising tones that had elicited a small but significant male advantage in our earlier studies [11], as well as with a rapid temporal auditory processing task that required participants to distinguish pairs of pure tones differing in amplitude envelope rise times by 60 ms. The latter contrast is devoid of segmental or spectral information which allowed us to assess temporal processing ability in its pure form. Processing of these types of auditory stimuli has been shown to be predictive of speech processing impairments associated with dyslexia [6]. The main questions of interest were whether (a) a sex difference would not just be replicated for the tonal contrast but could be observed for the temporal contrast as well, and (b) whether effects of sex and of higher cognitive abilities on the processing of the unfamiliar tonal contrast would be mediated by temporal auditory processing.

In addition, we also presented our participants with a Norwegian vowel contrast, to determine whether the male advantage is indeed confined to stimuli requiring processing of temporal, as opposed to spectral information. The non-native vowel contrast comprised minimal pairs of Norwegian words containing the vowel/i:/or/I/vs./y:/or/Y/, a contrast between a high front unrounded and rounded vowel which does not exist in most dialects of English. If the male advantage is indeed based on a superior temporal processing ability then we would expect to see a male advantage in discrimination of the tonal, but not the vowel contrast.

There is debate about whether the processing of speech sounds can be predicted by the processing of purely auditory temporal information [24] or whether diffeerent mechanisms and neural circuits underlie the processing of speech sounds vs. non-speech-related auditory analogues. Indeed, most studies on speech-sound processing have used synthesised contrasts in isolation or contrasts embedded in nonsense syllables. To determine whether the same effects of sex, temporal auditory processing ability and higher-order cognitive abilities can be observed for speech contrasts embedded in real language and for the corresponding auditory features in isolation, we presented participants with a non-speech analogue of the tonal contrast containing the crucial acoustic feature—the pure-tone pitch contour extracted from the Norwegian tones.

Finally, we were interested in also including a native speech sound contrast because previous research has suggested that while processing of native vs. non-native contrasts and their non-speech analogues results in similar performance on a behavioural level, only the processing of native speech sounds is associated with activation in Broca's area [25]. Given these putative differences in the neural mechanisms underlying the processing of native speech sounds vs. non-native speech sounds or non-speech sounds, it is important to examine whether the same factors predict individual differences in the processing of such stimuli. We therefore included a native contrast, the contrast between the English vowels/æ/and/ε/which were embedded in real words presented in noise to eliminate ceiling effects. All sound stimuli were presented in an AX-discrimination task requiring participants to judge whether the two members of a pair were the same or different.

To examine the link between temporal processing, speech-sound processing, and higher-order cognitive abilities, we measured non-verbal intelligence, verbal working memory capacity, and aspects of executive functioning. Psychometric measures of intelligence as well as span measures of verbal working memory capacity have been shown to predict speech processing under adverse conditions [21]. We also included the Simon Task to assess participants' inhibitory control [26], which may be important in tasks that require participants to focus on relevant acoustic dimensions while ignoring irrelevant ones. In the Simon Task, participants have to respond to one stimulus dimension (e.g., colour) while ignoring another prepotent dimension (e.g., spatial location).

Finally, to control for variables that are known to affect foreign language learning in various domains, we included questionnaires to assess prior experience with other foreign languages, musical ability, which has been linked to non-native phonological processing [27], [28], and handedness consistency, an indicator for the degree of inter-hemispheric connectivity [29], [30], an anatomical feature that has been implicated in successful learning of non-native consonants [31].

## Methods

### Participants

One hundred and twenty native speakers of British English (60 male and 60 female, mean age 24 years, range 18 to 61 years) participated in the sound processing and cognitive tasks. An additional ten native speakers of Norwegian (4 male and 6 female, mean age 21 years, range 20 to 22 years) participated in some of the sound processing tasks only. All participants reported no speech or hearing impairments. Participants provided written informed consent and received GBP 10.00 for participation in the study.

### Ethics Statement

The study had been approved by the Ethics Committee of the School of Social and Health Sciences of the University of Abertay Dundee.

## Materials

### Speech sounds

We selected eight pairs of monosyllabic Norwegian words comprising the vowel contrast (henceforth ‘vowel condition’) and eight pairs of bisyllabic Norwegian words comprising the tonal contrast (henceforth ‘tonal condition’). Within each type of contrast, four of the word pairs contained short the vowels/I/and/Y/(mean length 67 ms and 64 ms, for vowels and tones, respectively), and the remaining four word pairs contained the long vowels/i:/and/y:/(mean length 150 ms and 187 ms, for vowels and tones, respectively). In both conditions, corresponding short and long vowel pairs were matched for initial phoneme. In the tones, vowel length was varied on the first syllable, which was always the stressed syllable. A 2 (Vowel Length: short vs. long)×2 (Contrast:/i:/or/I/vs./y:/or/Y/) ANOVA on the vowel durations in the vowel condition yielded a main effect of vowel, *F*(1,28) = 18.31, *p*<.001, and no effect of Contrast (*p*>.9). The same type of ANOVA conducted on the word durations for the bi-syllabic tonal stimuli yielded no significant effects. This shows that the vowel contrast and the tonal contrast were not confounded with variation in the duration of the vowels and words.

For the native/æ/-/ε/vowel contrast (henceforth ‘English vowel condition’), we selected eight monosyllabic English words, which did not systematically differ in vowel length. To maintain comparability with the Norwegian stimuli, two word pairs were always matched for initial phoneme. All words used in the experiment are listed in Table S1. To capture the within-category variability characteristic of natural speech, two different instances of each word were selected from a set of six instances recorded by a balanced bilingual male speaker of Norwegian and English (JCT). All instances were embedded in the same carrier phrase and were recorded at a sampling rate of 441 kHz. The target instances were selected based on similarities in duration and intonation. The English words were then combined with a recording of naturalistic cocktail noise from the file cafeteriaflac on www.freesound.org using Praat [32]. For each speech contrast, within-category variants of the same word formed the ‘same’ pairs and minimal pairs comprising the different categories of the tonal or vowel contrast of interest formed the ‘different’ pairs. The materials also contained the English/s/-/θ/and the Norwegian/ç/-/∫/consonant contrasts but because performance for those stimuli was at ceiling with little variability the results will not be reported here.

### Non-speech sounds

Stimuli for the temporal processing condition (henceforth ‘amplitude condition’) were sinusoidal carrier waves at 250 Hz with an overall duration of 600 ms, faded out over 50 ms. The onset of the amplitude envelope was faded in with rise times to reach maximum amplitude at 0 ms, 10 ms, 20 ms, 30 ms, 60 ms, 70 ms, 80 ms and 90 ms. For the AX task, ‘different’ trials comprised pairs of sounds differing in rise times by 60 ms (eg 0 ms vs. 60 ms or 10 ms vs. 70 ms etc), centered around 45 ms, a value which has been reported as the category boundary between ‘bowed’ and ‘plucked’ sounds [33]. Thus, as in the speech sounds, this manipulation introduced a category contrast. The non-speech equivalent of the Norwegian tonal contrast (henceforth ‘pitch-contour condition’) comprised a sine wave with a contour extracted from the fundamental frequency modulation of the Norwegian tones. These stimuli contained no other information but the pitch contour of the Norwegian tones.

All five types of sound stimuli were combined into 64 pairs, half of which were ‘same’ and half ‘different’ trials.

### Cognitive measures

We measured non-verbal intelligence using Cattell's Culture Fair Intelligence Test [34], verbal working memory capacity using the Reading Span Test [35], and executive functioning using the Simon Task [26].

#### Culture Fair Intelligence Test

We used Scale 3, Form A [34] which contains four sets of abstract geometrical multiple-choice pattern completion problems with allotted solution times per set varying between 25 and 4 minutes. Two of the problem sets (‘Series’ and ‘Matrices’) involved selecting an abstract geometric stimulus (from six alternatives) to complete a series or pattern (matrix). One problem set (‘Classification’) required the participant to identify which two out of five stimuli were alike in some way (i.e., different from the other three). The last problem set (‘Conditions/Topology’) required the participant to select a stimulus (out of five alternatives) that matched a template with respect to the placement of a dot among geometric forms. This test was analysed using the provided scoring template to determine the total number of correct items for each participant.

#### Reading Span Test

We used the original version developed by Daneman and Carpenter [35] consisting of 70 sentences. Participants were are asked to read aloud sets of sentences and to recall the last word of each sentence at the end of each set Set size increased from 2 to 5. The final score was computed by counting the total number of correctly recalled sentence-final words (out of 70), a measure that has been shown to provide greater diagnostic value than a span measure [36].

#### Simon Task

Participants had to respond with left or right button presses to the colour of a square (red vs. blue) ignoring the location of the square on the monitor (left vs. right). The visual stimuli were presented using E-prime 20 (Psychology Software Tools, Pittsburgh, PA) on a 15-inch monitor. Each trial started with a fixation cross presented at the middle of the screen for the duration of 800 ms. After a blank interval of 250 ms, a red or blue square appeared on the left or right of the screen and remained there for 1000 ms if there was no response. Participants were instructed to press the ‘1’ key if they saw a red square and the ‘ = ’ key when they saw a blue square. Assignment of colours to keys was counterbalanced across participants. Timing began at onset of the visual stimulus; presentation was terminated when the response occurred. After eight practice trials participant completed 28 trials, half of which presented the square on the same side as the associated response key (congruent trials) and half on the opposite side (incongruent trials). The difference between the reaction times for incongruent and congruent trials (henceforth: Simon Cost) is a measure of the cost of suppressing prepotent irrelevant information.

#### Edinburgh Handedness Inventory (EHI)

The EHI [37] was used to assess degree of handedness consistency. Participants were asked to rate how often they carry out 10 manual actions (e.g., striking a match, opening a jar) with the right or left hand. Scores are assigned on a scale from −100 (extreme left-handedness) to 100 (extreme right-handedness). Handedness consistency was determined using the absolute values of the EHI scores, with higher values indicating more consistent handedness corresponding to less inter-hemispheric connectivity.

#### Musical Ability and Language Background

To assess musical ability and prior experience to foreign languages participants were asked to complete a Musical Ability questionnaire and a Language Background questionnaire. On these questionnaires, participants rated their musical ability and their level of reading, writing, speaking and comprehension for each foreign language on a scale from 1 (very poor) to 6 (excellent). We opted for self-ratings of these abilities in order to minimise participant burden associated with multiple assessments. If participants had studied more than one foreign language, the language with the higher self-rating was coded as L2 and the language with the lower self-ratings was coded as L3. If participants had studied only one foreign language the ratings for L3 were set to 0. Note that some participants had studied more than two foreign languages; hence, we also coded the total number of studied languages.

### Procedure

After giving written, informed consent, participants first completed the Musical Ability and Language Background questionnaires. They then received three blocks, each containing two auditory conditions and one of the cognitive tasks (ie, the Culture Fair Intelligence Test, the Reading Span Test and the Simon Task). Thus, the cognitive tests were interspersed with the sound discrimination tasks. Order of tasks was randomized with two exceptions: The amplitude condition was always presented first to ensure that temporal processing was not affected by potential order effects; the pitch-contour condition was always presented last to ensure that this task did not prime discrimination of the Norwegian tonal contrast. The entire session lasted about 90 minutes.

In the AX tasks, participants were presented with the two sound stimuli over Sennheiser headphones. The sound stimuli within a pair pair were separated by an inter-stimulus interval of 200 ms; the inter-trial interval was 500 ms. The instructions provided participants with some information about the location of the crucial contrast, i.e., whether it was to be expected at the beginning or in the middle of the sound stimulus or whether it pertained to the pitch of the sound. Participants were asked to press the ‘S’ key if they perceived the sounds to be the same and the ‘D’ key if they perceived them to be different. The 64 test trials were preceded by 8 practice trials during which participants received feedback. No feedback was given for the test trials

The native speakers of Norwegian completed only the Norwegian vowel and tonal conditions and the non-speech pitch-contour condition, to check whether native speakers could indeed easily identify the presented contrasts.

## Results

Three men and 3 women over the age of 39 years were excluded from further analyses to minimize potential effects of age-related decline in peripheral and central auditory processing [38]. In addition, two participants failed to rate their foreign language proficiency and had therefore be excluded from those analyses that involved this variable. Participants' performance on the AX-tasks was converted into *A′*, a sensitivity measure that corrects for differences in response bias *A′* scores range from 0 to 1, with 05 corresponding to chance [39]. Table 1 shows performance in each condition for male and female English and Norwegian speakers. Planned comparisons revealed that Norwegian native speakers outperformed the English native speakers in discriminating the two Norwegian contrasts and the extracted pitch contour, all *t*s>2.80, all *p*s<.01. These values were significant after Bonferroni-correction, confirming that the English speakers indeed experienced more difficulties than native speakers when discriminating the Norwegian contrasts.

**Table 1:** Mean *A′* and standard deviations in parentheses for the auditory discrimination tasks in native English and native Norwegian speakers.

Listener native language	Amplitude	English vowels	Norwegian vowels	Norwegian tones	Pitch contours
English:					
overall	0.83 (0.08)	0.93 (0.04)	0.90 (0.05)	0.84 (0.08)	0.83 (0.08)
men	0.85 (0.07)	0.93 (0.04)	0.90 (0.06)	0.85 (0.07)	0.85 (0.08)
women	0.81 (0.09)	0.92 (0.04)	0.89 (0.05)	0.82 (0.09)	0.81 (0.07)
Norwegian:					
overall			0.95 (0.04)	0.93 (0.04)	0.92 (0.03)
men			0.94 (0.03)	0.93 (0.05)	0.91 (0.04)
women			0.95 (0.05)	0.94 (0.04)	0.92 (0.03)

For the native English speakers, a 5 (Sound Condition)×2 (Sex) mixed-type ANOVA revealed a main effect of condition, *F*(4,448) = 76.6, *p*<.0001, a main effect of sex, *F*(1,118) = 7.7, *p* = .006, as well as a significant interaction between the two factors, *F*(4,448) = 3.2, *p* = .013. Post-hoc comparisons using Bonferroni-corrected t-tests revealed that the performance in the English vowel condition was significantly better than in the Norwegian vowel condition, which, in turn, was better than in the amplitude condition, the Norwegian tonal condition and the pitch-contour condition, all *t*s(113)>6.7, all *p*s<.001 Performance in the amplitude condition, the Norwegian tonal condition and the pitch-contour condition did not differ significantly from each other. The interaction between Sound Condition and Sex was due to a significant male advantage in the pitch-contour condition, *t*(112) = 3.0, *p* = .003, the amplitude condition, *t*(112) = 2.7, *p* = .008, and the Norwegian tones: *t*(112) = 2.0, *p* = .047), but not the English vowels, *t*(112) = 1.4, *p* = .160, nor the Norwegian vowels, *t*(112) = 0.02, *p* = .87.

Table 2 presents the correlations between *A′* scores in all discrimination tasks All correlations were significant after Bonferroni correction. Indeed, a principal component analysis revealed only one factor with an eigenvalue greater than 1 suggesting that performance in all the tasks relied to some extent on shared processing components. Table 3 shows the correlations between all cognitive variables and sex (coded as a dummy variable with 0 for men and 1 for women). The predictor variables were largely uncorrelated except for correlations between the L2 and L3 self ratings and total number of languages studied. We also found an association between performance on the Culture Fair intelligence test and the Reading Span test. Although it did not reach significance after Bonferroni-correction, such a link is consistent with previous findings [40], [41] and has been attributed to a shared executive component underlying both tasks.

**Table 2:** Zero-order correlations between performance in the sound conditions Asterisks indicate significance after Bonferroni-correction.

	2	3	4	5
1 Amplitude	.31^**^	.31^**^	.46^**^	.34^**^
2 English vowels		.45^**^	.34^**^	.27^**^
3 Norwegian vowels			.46^**^	.45^**^
4 Norwegian tones				.60^**^
5 Pitch contours				

Note: ** p<.001.

**Table 3:** Zero-order correlations between predictor variables used in the study Asterisks indicate significance after Bonferroni-correction.

	2	3	4	5	6	7	8	9
1 CFIQ	.25	−.09	.08	.09	.02	.10	−.07	−.09
2 ReadSpan		−.06	.21	.03	.20	.10	−.03	.06
3 SimonCost			.17	.08	.08	.00	.19	−.11
4 Ltotal				.27	.70**	−.07	−.02	.08
5 L2 rating					.52**	.15	.02	.07
6 L3 rating						.00	.00	.10
7 MusRating							−.08	.07
8 HandCons								−.16
9 Sex								

Note: ** p<.001.

Table 4 shows the results of multiple regression analyses with performance on the discrimination tasks as criterion variables and the cognitive measures as well as sex as predictor variables. These analyses revealed a significant effect of participant sex over and above all other variables in both the amplitude condition and the pitch-contour condition (for group means refer to Table 1), which confirmed the predicted male advantage in tasks requiring temporal processing. For discrimination performance in the English vowels presented over noise, there was a significant effect of Simon Cost. This effect was also marginally significant for the Norwegian vowels. Musical ability uniquely contributed to discrimination performance with the Norwegian sounds and the extracted pitch contours. The regression analysis for the pitch-contour condition additionally showed a negative effect of proficiency in the highest-rated foreign language. Finally, there was a unique effect of non-verbal intelligence in the amplitude condition and the pitch-contour condition, such that individuals who scored higher in the Culture Fair intelligence test showed better discrimination performance. Note that in both conditions, the contribution of non-verbal intelligence to task performance was independent of the effect of sex.

**Table 4:** Standardised correlation coefficients and proportion of variance accounted for by regression analyses with sound stimuli as criterion variables and cognitive measures and sex as predictor variables.

	amplitude	English vowels	Norwegian vowels	Norwegian tones	pitch contours
CFIT	.25**	.11	.13	.18^†^	.20*
ReadSpan	.11	.14	.12	.08	.00
SimonCost	−.10	.24*	.20	.00	.03
Ltotal	−.07	−.04	.07	.13	.13
L2Rating	−.02	.00	.05	−.06	−.27**
L3Rating	.22	.18	.01	−.03	.07
MusRating	.08	.03	.21*	.24*	.40***
HandCons	.01	.11	−.05	−.01	.13
Sex	−.26**	−.12	.00	−.19*	−.25**
Adj R^2^	.15	.09	.08	.09	.24
F(9,102)	3.10**	2.18*	2.05*	2.15*	4.96***

Note: CFIT - Culture Fair Intelligence Test, ReadSpan – Reading Span test, SimonCost – incongruent minus congruent RT in the Simon test, Ltotal – number of foreign languages ever learned, L2Rating – mean self-rating for reading, writing, speaking and comprehending in the first L2, L3Rating - mean self-rating for reading, writing, speaking and comprehending in the second L2 (if there was no L3 this variable was set to 0), MusRat – self rating of musical abilities, HandCons – consistency of handedness as assessed by the Edinburgh Handedness Inventory, Sex – dummy-coded with 0 for men and 1 for women ***p<.001, **p<.01, *p<.05,†p<.1.

The next set of analyses aimed to tease apart the roles of cognitive factors and temporal processing in predicting individual differences in the discrimination of the various sounds. If temporal processing is causally linked to measures of non-verbal intelligence, as well as to the ability to process sound stimuli containing rapid temporal changes, then the effect of non-verbal intelligence should disappear once temporal processing is added to the multiple regression model. If, on the other hand, both temporal processing and non-verbal intelligence have independent effects on sound discrimination, then both of these variables should have significant effects in the model. To decide between these alternatives, performance in the amplitude condition was added to each multiple regression model. The results, which remained virtually unchanged when participant age was added to the regression models, are displayed in Table 5.

**Table 5:** Standardised correlation coefficients and proportion of variance accounted for by regression analyses with sound stimuli as criterion variables and cognitive measures, gender and performance in the amplitude condition as predictor variables.

Predictor variable	English vowels	Norwegian vowels	Norwegian tones	Pitch contours
CFIT	.04	.05	.08	.14
ReadSpan	.11	.08	.04	−.03
SimonCost	.26**	.23*	.04	.05
Ltotal	−.02	.09	.16	.15
L2Rating	.00	.05	−.06	−.26**
L3Rating	.12	−.06	−.12	.01
MusRating	.01	.18*	.21*	.38**
HandCons	.11	−.05	−.01	.13
Sex	−.04	.08	−.09	−.18*
Amplitude	.27**	.31**	.39***	.23*
Adj R^2^	.14	.15	.21	.28
F(10,107)	2.83**	2.97**	3.94***	5.35***

Note: CFIT - Culture Fair Intelligence Test, ReadSpan – Reading Span test, SimonCost – incongruent minus congruent RT in the Simon test, Ltotal – number of foreign languages ever learned, L2Rating – mean self-rating for reading, writing, speaking and comprehending in the first L2, L3Rating - mean self-rating for reading, writing, speaking and comprehending in the second L2 (if there was no L3 this variable was set to 0), MusRat – self rating of musical abilities, HandCons – consistency of handedness as assessed by the Edinburgh Handedness Inventory, Sex – dummy-coded with 0 for men and 1 for women ***p<.001, **p<.01, *p<.05.

Indeed, the effect of non-verbal intelligence on pitch-contour discrimination was no longer significant when performance in the amplitude condition was added to the model, whereas the effects of L2 self-rating, musical ability and sex remained significant. This suggests that effects of non-verbal intelligence on sound discrimination are due to temporal processing, which also contributes to non-verbal intelligence [19], [42], [43]. In contrast, the effects of self-rated musical ability and of Simon Cost on sound discrimination appear not to be linked linked with temporal processing.

To confirm that the effect of non-verbal intelligence on sound discrimination was indeed mediated by temporal processing we performed a set of mediation analyses employing bootstrapping to estimate the 95% confidence intervals of the indirect effect using the procedure introduced by Hayes and Preacher for multiple predictor variables (SPSS-macro MEDIATE, downloaded from http://www.afhayes.com/spss-sas-and-mplus-macros-and-code.html). The relative indirect effect is deemed to be statistically significant at *p*<.05 if these confidence intervals do not include zero. Table 6 lists the confidence intervals of the omnibus indirect effect, and for the individual predictor variables. These results indicate significant relative indirect effects. Three results of the mediation analyses are noteworthy: First, given that non-verbal intelligence ceased to have a significant direct effect once temporal auditory processing was added to the regression model, the results suggest that the effect of non-verbal intelligence on sound discrimination ability was fully mediated by temporal processing. The analyses also revealed indirect effects in the English and Norwegian vowel conditions and the Norwegian tonal condition where there were no significant direct effects of non-verbal intelligence. This finding may at first glance seem paradoxical if one assumes that mediation analyses are used to test whether the direct effect of variable X on variable Y is mediated by an effect of X on some mediator M, which, in turn, is affecting Y. However, the same statistical procedure can be used to show that a variable M (temporal processing, in our case) can affect both X (non-verbal intelligence) and Y (sound processing); for a more detailed explanation, see [44]. If interpreted in this way, the mediation analyses support the hypothesis that temporal processing contributes to both non-verbal intelligence and speech-sound processing ability even in cases where the link between the two latter variables did not reach statistical significance in the multiple regression analyses.

**Table 6:** 95% confidence intervals for the indirect effects mediated by the temporal processing task (amplitude condition) as well as the omnibus indirect effect.

Pred var.	Condition							
	English vowel Contrast		Norwegian vowel contrast		Norwegian tonal contrast		Pitch contour contrast	
	Lower limit	Upper limit	Lower limit	Upper limit	Lower limit	Upper limit	Lower limit	Upper limit
CFIT	**.0000**	**.0012**	**.0000**	**.0020**	**.0002**	**.0035**	**.0000**	**.0022**
RS	−.0001	.0006	−.0002	.0009	−.0003	.0019	−.0002	.0011
SC	−.0001	.0000	−.0001	.0000	−.0002	.0000	−.0001	.0000
LTot	−.0045	.0023	−.0074	.0027	−.0136	.0060	−.0080	.0036
L2	−.0026	.0022	−.0044	.0031	−.0087	.0063	−.0053	.0035
L3	−.0005	.0062	−.0004	.0097	−.0011	.0189	−.0008	.0114
Mus.	−.0007	.0025	−.0010	.0036	−.0021	.0070	−.0012	.0042
Hand.	−.0001	.0001	−.0001	.0001	−.0002	.0003	−.0001	.0001
Sex	**−.0118**	**−.0008**	**−.0186**	**−.0014**	**−.0358**	**−.0040**	**−0217**	**−.0007**
Omn.	**.0040**	**.0605**	**.0064**	**.0994**	**.0190**	**.1829**	**.0040**	**.1139**

Confidence intervals not including 0 are marked in boldface; 0000 represents an exceedingly small positive value. Note: CFIT - Culture Fair Intelligence Test, RS – Reading Span test, SC – incongruent minus congruent RT in the Simon test, Ltot – number of foreign languages ever learned, L2 – mean self-rating for reading, writing, speaking and comprehending in the first L2, L3 - mean self-rating for reading, writing, speaking and comprehending in the second L2 (if there was no L3 this variable was set to 0), Mus. – self rating of musical abilities, Hand. – consistency of handedness as assessed by the Edinburgh Handedness Inventory, Sex – dummy-coded with 0 for men and 1 for women, Omn. – omnibus indirect effect.

Second, the mediation analyses revealed an indirect effect of sex mediated by temporal auditory processing in all conditions, even in those conditions where sex did not have a significant direct effect. This suggests that when a male advantage in the processing of speech sounds is found, it is mediated by a male advantage in temporal processing. However, the fact that the direct effect of sex remained significant in the pitch-contour condition after temporal processing was added to the model suggests partial mediation: In addition to temporal processing, the tested males may have had an advantage in some other skill that also affects pitch-contour discrimination. Finally, the effects of self-rated musical ability, Simon Cost and self-rated L2 proficiency on sound discrimination performance were direct effects and were not mediated by temporal processing^3^.

## Discussion

This study examined to what extent temporal processing ability (as measured by discrimination of amplitude envelope onset rise times), and measures of various aspects of cognitive functioning explain individual differences in non-native speech-sound discrimination. We were also interested to see whether the same predictors would explain performance in a non-speech analogue of a non-native tonal contrast and in a native contrast presented over noise, to clarify whether any determinants of individual differences are specific to speech vs. non-speech sound processing, as well as to non-native vs. native speech sound processing.

### Sex differences

We found a male advantage in temporal processing as well as evidence from mediation analyses that sex differences in speech-sound processing are mediated by individual differences in temporal processing. By demonstrating the relevance of sex differences in temporal processing to speech processing, our findings add to previous work documenting a male advantage in temporal order judgments [14], [15] and temporal discrimination tasks [16]. Note that in the pitch-contour condition, there was evidence of partial mediation of the effect of sex by temporal processing, which suggests that other factors might also have contributed to the sex difference in that condition. Thus, future studies will have to explore whether other components of speech-sound processing, such as spectral processing, might also show a male advantage.

What mechanisms might be responsible for the observed sex difference? In a perceptual training study, Golestani, Molko Dehaene, LeBihan and Pallier [45] demonstrated that faster learning of non-native speech sounds involving rapid spectral changes was associated with differences in brain anatomy. Specifically, faster learning was linked to larger overall white matter volumes in left Heschl's gyrus and increased degree of left vs. right asymmetry in white matter density in auditory cortex. Increased white matter volume may indicate greater myelination resulting in more rapid neural transmission, which is crucial for perception of rapid temporal changes. It might also be due to a greater number of white matter fibres connecting language regions within and between cortical hemispheres, such as fibres connecting the auditory cortex with anterior and posterior language regions. Given that for these perceptual learning tasks performance at the outset is highly correlated with speed of learning [46] it is reasonable to speculate that similar anatomical changes might also distinguish individuals who perform better in perceptual discrimination tasks when presented with non-native speech sounds for the first time, as in the present study. There is also evidence for a negative correlation between white matter volume and variability in isochronous tapping in the sub-second range [47] suggesting that white matter volume can be implicated in rapid temporal processing in other modalities as well. Comparisons of male and female brain anatomy and cytoarchitecture have revealed larger white to grey matter ratios in men than in women, and less white matter asymmetry between hemispheres in women (48), which might account for the observed sex differences in temporal processing. Gur et al [48] suggested that the maintenance of grey matter volume consisting of somatodendritic tissue—responsible for computation—at the relative expense of myelinated connective tissue—responsible for information transmission—may be a reasonable evolutionary strategy for dealing with the smaller cranial volumes of females, where transmission occurs over relatively shorter distances than in males. It should be noted, however, that increased ability to imitate speech sounds has been linked to enhanced grey matter volume in a left fronto-parietal network [17] indicating that future research is needed to tease apart how specific aspects of the neuro-anatomical substrate are linked to specific task components associated with perceiving and producing non-native speech sounds.

### Effects of non-verbal intelligence

We also found that the effect of non-verbal intelligence on speech processing, most notably on the processing of pitch contours, was mediated by temporal processing. We suggest that our findings point towards a model in which temporal processing contributes to both non-verbal intelligence as well as speech processing. This is in line with previous findings of a link between temporal processing and non-verbal intelligence [19], [42], [43]. One question that arises is why are there no sex differences in general intelligence given a sex difference in temporal processing and a link between temporal processing and intelligence? Indeed, mean sex differences in general intelligence have generally proven to be elusive [49] despite sex differences in reaction times [50] and temporal processing [16]. However, a review of research on sex differences in various timed tests reveals that men are faster in reaction time and finger tapping tests while women are faster in naming and symbol copying, with neither sex outperforming the other in general intelligence [51]. Thus, while reaction time and temporal processing appear to explain some of the variance in general intelligence, other performance components are also bound to play a role and these components do not necessarily favour men.

### Processing of speech vs. non-speech sounds

Our study allowed us to compare the discrimination of speech sounds (ie, Norwegian tones) and their non-speech acoustical analogues (ie, the extracted pitch contours). Overall performance did not differ between the speech and the non-speech conditions, which suggests that participants were able to identify the pitch contours as the relevant feature when comparing the words that comprised Norwegian tonal minimal pairs. Moreover, we found direct effects of non-verbal intelligence, sex and musical ability in both conditions suggesting similar effects of these variables across speech and non-speech stimuli. Thus, our findings are in line with studies showing similar mechanisms and underlying neural substrates being activated by non-native contrasts and their non-speech analogues [25].

### Effects of foreign language experience

Foreign language experience was assessed by self-ratings of reading, writing, listening and speaking ability in each of the previously studied languages. In general, the participants' self-rated proficiency in previously studied foreign languages had no effect on their ability to discriminate the presented speech sounds. Note that none of our participants had been exposed to a language with tonal contrasts so that transfer of prior experience with this feature was not possible. The only effect of prior experience with previously studied languages emerged in the pitch contour condition where discrimination performance was negatively linked to self-ratings of proficiency in the highest rated L2: The higher participants rated their proficiency in the foreign language they knew best the more difficulty they had discriminating the non-speech pitch contours. This is an unexpected finding for which we do not have a satisfactory explanation. Further replication is required before speculating about potential mechanisms behind such a link.

### Effects of executive functioning

It may seem that our conclusion that temporal processing underlies both non-verbal intelligence as well as non-native speech processing in the tonal conditions differs from studies of native speech processing under adverse conditions, where temporal acuity and psychometric intelligence have been found to make independent contributions [21]. However, we also found independent effects of a cognitive variable, Simon Cost, and of temporal auditory processing on the discrimination of both the native and non-native vowel contrasts. We suggest that the specific mechanisms by which temporal processing and cognitive abilities affect speech processing depend on the various task demands and the cognitive measures employed: When processing native speech sounds under adverse conditions listeners need to identify familiar, but degraded, phonemic categories. This requires the ability to inhibit noise and to focus attention on the signal; such cognitive control mechanisms are well captured by tests of executive functioning like the Simon Task. The observed positive link between Simon Cost and the processing of a native vowel contrast under noise suggests that it is the executive functioning component inherent in cognitive assessments [52] that predicts native speech processing under adverse conditions, and that this contribution is independent of the effect of temporal (and, perhaps, spectral) acuity. In other words, the effects of cognitive measures found in studies of native speech processing [21], [22], [23] may have been mainly due to the executive components associated with efficient noise inhibition and attention allocation, a link that may in future research be better captured by more direct measures of executive control.

However, when discriminating non-native, unfamiliar speech sounds, listeners need to identify the perceptual dimensions along which to re-structure familiar phonemic categories or to create new phonemic categories. In such situations, we would expect perceptual acuity to play a larger role than executive functioning. Indeed, for the tonal contrasts, we did not find independent effects of Simon Cost but of temporal acuity and self-rated musical ability. This may be due to the pitch contours, which extended over several milliseconds, being quite easy to notice but not as easy to distinguish. We speculate that in tasks where the relevant acoustical information is readily available, the ability to inhibit irreleveant information is taxed to a lesser extent, although we cannot exclude the possibility that inhibition of irrelevant segmental information is needed when trying to discriminate between tonal contours. Still, in such situations, perceptual abilities related to temporal processing should play a larger role as they provide the raw data upon which category formation can work.

Executive functioning may be more important for non-native phonemes that are shorter in duration and therefore not as easily identifiable. Specifically, when processing the Norwegian words containing the vowel contrast participants had to allocate their attention to that specific segment and inhibit information from other segments. The generally successful discrimination performance with this contrast suggests that our English-speaking participants had little difficulty forming the relevant categories, not least because one member of the minimal pair, the vowel/i/, could be assimilated to a familiar category in their native language [53]. Thus, for these stimuli, the challenge may have been to inhibit the information associated with the rest of the word, and to focus their attention on the one crucial segment (phoneme), a requirement that might be responsible for the positive link with executive functioning in this condition.

### Musical ability and non-native speech processing

Our findings also showed positive associations between self-ratings of musical ability and discrimination of the Norwegian vowels, tones, and extracted pitch contours. This is in line with previous evidence that musical training and musical ability are related to superior non-native tonal processing [54], [55]. There is some inconsistency as to whether musical ability and musical training also affect non-native phonological processing: Some studies have shown such a link [27], [28], whereas others have not [54]; for a detailed review see [56]. Our study supports the existence of this link for the discrimination of non-native vowels for which spectral acuity plays an important role. The inconsistencies between various studies might be due to the differences in the phonological contrasts tested as well as to differences in the measures of musical ability used. Whereas some studies, like the present one, have used musicality self-ratings [28], others have measured musical ability using objective tests such as chords analysis, pitch change detection and tonal memory [28], [54] or have compared musicians and non-musicians using years of musical training as the distinguishing criterion [27], [57]. These measures may tap into slightly different aspects of musicality. Interestingly, Slevc and Miyake [28] did not find an effect of musical ability self-ratings on receptive and productive phonology measures in learners at later stages of L2 acquisition, whereas in our study self-ratings were predictive of the ability to discriminate non-native speech sounds. Because our participants had encountered these speech sounds for the first time this finding suggests that musical ability may play a larger role at the outset of learning than at later stages.

It has been suggested that the positive association between experience with music and L2 learning might be due to superior attentional control attained through musical training, which then benefits both musical and linguistic processing [58]. Our data do not support this idea as we did not find a correlation between musical ability and inhibitory control as measured by the Simon task. Instead, our data are more in line with the idea of transfer of abilities relevant to music processing to the linguistic domain. In this study, these abilities were predominantly related to performance with stimuli requiring processing of pitch and spectral information. However, there are also suggestions that exposure to music improves general temporal processing abilities [57]. The finding that, in this study, self-rated musical ability was not related to temporal auditory processing in the amplitude condition, but rather to performance in conditions that required processing of pitch and spectral information, is in line with suggestions that music predominantly requires processing of pitch and spectral information [59], and that temporal changes relevant to music occur on a slower time scale than temporal changes relevant to the discrimination of speech segments [60]. However, given that we did not define musical ability to our participants it is possible that their ratings were more influenced by their self-assessment of pitch and spectral processing ability (e.g. being able to reproduce a note or a harmonic interval or to hold a tune) than temporal processing ability. As the present study did not include an objective test of the participants' ability to process pitch and spectral information, we can at this point only speculate that the observed link between musical ability and non-native speech-sound processing might indeed be due to superior pitch and spectral processing. Further studies will have to show to what extent temporal vs. spectral sensitivities underlie musical and linguistic aptitude, to what extent temporal and spectral processing abilities can be improved by musical training and to what extent such improved temporal and spectral processing abilities can indeed be transfered to various aspects of language learning.

In sum, our findings suggest that temporal auditory processing plays a crucial role in non-native speech-sound processing, and may be the mechanism mediating the effects of non-verbal intelligence, as well as sex, on speech-sound discrimination. We also found that other variables like musical ability can affect non-native speech-sound processing. In closing, we would like to mention several caveats of the study that suggest avenues for further research. One important caveat is associated with the specific characteristics of the AX discrimination task employed in this study: To perform a judgment about whether two successively presented sound stimuli are the ‘same’ or ‘different’ participants must retain the first stimulus in phonological short-term memory in order to compare it with the second one. While the ISI of 200 ms used in this study was chosen to minimize memory demands, we nonetheless cannot rule out that phonological short-term memory capacity is an important constraint on performance in AX tasks. Consequently, a proportion of the variance accounted for by the temporal auditory processing task might, in fact, be associated with phonological short-term memory capacity, simply because temporal processing was measured with the same AX discrimination task that was also used in the other sound-processing conditions. To clarify which effects are associated with temporal auditory processing and which effects are due to memory demands, future studies of individual differences in non-native speech-sound processing should employ a wider variety of tasks and dependent variables, such as ERPs, to measure mismatch negativity in oddball detection paradigms. As suggested above, future studies should also measure spectral processing acuity in addition to temporal processing acuity to elucidate the relative contribution of both of these perceptual mechanisms to non-native speech processing.

Another caveat is associated with the fact that we only tested one specific pairing of native and non-native speech sounds, English and Norwegian. While this choice was determined, among other things, by considerations of accessibility and feasibility, it nonetheless constitutes a legitimate research design as the non-native contrasts tested were not present in the native language of the participants. Still, for future research it will be important to increase the generalizability of these findings by testing other native – non-native language pairings. These caveats notwithstanding, the present findings show that adults presented with novel phonemic and tonal categories in non-native speech flexibly deploy different perceptual and cognitive mechanisms in ways that are dependent on the specific characteristics of the sounds. Thus, in accordance with the variable features of speech sounds, individual differences are likely to be explained by different perceptual and cognitive abilities of which temporal auditory processing is only one.

## Supporting Information

Table S1 **Speech stimuli used in the experiments.**
(DOCX)
