# Supplementary material for: Individual Differences in the Discrimination of Novel Speech Sounds: Effects of Sex, Temporal Processing, Musical and Cognitive Abilities
Source: PLoS One. 2012 Nov 5;7(11):e48623. doi: 10.1371/journal.pone.0048623 (PMC3489723; doi:10.1371/journal.pone.0048623)
Supplement: Table S1 — Speech stimuli used in the experiments. (DOCX) [file pone.0048623.s001.docx]

S1: Speech stimuli used in the experiments

| Contrast category | Short vowel | Long vowel |
| --- | --- | --- |
| Norwegian  tonal  contrast | Bøtter [name] – bøtter [buckets]  gullet [gold] – gulle [to fluke]  legget [ID (slang)] – legge [to put]  rakket [dog] – rakke [to botch] | bøter_1_ [fines] – bøter_2_ [to repent]  gulet [wind] – gule [yellow]  läget [state (slang)] – lege [GP]  raket [wreck] – rake [rake] |
| Norwegian  vowel  contrast | rykk [pull] – rikk [budge]  syll [joist] – sild [herring]  mytt [molted] – mitt [mine]  lynn [to soften] – lind [lime tree] | ryk [to smoke] – rik [rich]  syl [awl]– sil [sieve]  myt [to moult] – mit [mite]  lyn [lightning] – lin [linen] |
| English  vowel  contrast | bad – bed, bat – bet  pan – pen, pat – pet  Dan – den, tan – ten  mat – met, man – men | |

Note: Words in brackets are the English translations. For Norwegian tonal contrasts the first word of the minimal pair is tone 1 (rising) and the second tone 2 (falling-rising).
